# Bordetella pertussis, an agent not to forget: a case report

**DOI:** 10.1186/1757-1626-2-128

**Published:** 2009-02-06

**Authors:** Natália Melo, Ana Catarina Dias, Lara Isidoro, Raquel Duarte

**Affiliations:** 1 Centro de Diagnóstico Pneumológico de Vila Nova de Gaia, Vila Nova de Gaia, Portugal; 2 Hospital de São João. Serviço de Pneumologia, Porto, Portugal; 3 Centro Hospitalar de Vila Nova de Gaia, Vila Nova de Gaia, Portugal; 4 Faculdade de Medicina, Universidade do Porto, Porto, Portugal

## Abstract

**Introduction:**

In the past, pertussis affected particularly children under 6 years of age, but recent trends show that there is a shift toward the older age group. The clinical presentation can be atypical in the adolescent age group, and the disease is often misdiagnosed.

**Case presentation:**

We present a case of an 11-year-old male patient oriented to our unit with anorexia, weight loss and persistent cough with nocturnal paroxysms for 4 weeks. He also reported occasional wheezing and chest tightness. He denied fever, chills, myalgia, sore throat, or rhinorrhea. The patient presented to his primary care physician 1 week prior with the same complaint and was treated with amoxicillin and ebastine. Facing the persistence of the complaints he was oriented to our unit in order to exclude tuberculosis. Further study confirmed *Bordetella pertussis *infection and he started clarithromycin (15 mg/kg/day for 14 days). The patient's symptoms resolved after two weeks. Two of the patient's family members have developed symptoms of *Bordetella pertussis *infection and were treated after convenient study.

**Conclusion:**

Cough is one of the most common complaints among children and its causes are multiple. Active immunization and early diagnosis are crucial in the management of pertussis.

## Introduction

In the past, pertussis affected particularly children under 6 years of age, but recent trends show that there is a shift toward the older age group [1]. In Western countries, approximately 10 to 12% of all pertussis cases occurred in persons over 15 years of age [2]. The anticipation and early diagnosis of these cases is important because the affected adolescents and adults act as reservoirs of the disease to the vulnerable population of infants, for whom the disease can be life threatening. The clinical presentation can be atypical in the adolescent age group, and the disease is often misdiagnosed. With the availability of polymerase chain reaction and serology, the disease can be diagnosed even later in the disease course.

## Case presentation

An 11-year-old male patient presented anorexia, weight loss and persistent cough with nocturnal paroxysms for the previous 4 weeks. He also reported occasional wheezing and chest tightness. He denied fever, chills, myalgia, sore throat, or rhinorrhea. The patient presented to his primary care physician 1 week prior with the same complaint, and was treated with amoxicillin, ebastine and bronchodilator therapy. The patient's symptoms did not improve with this regimen. The cough became more frequent, sometimes emetizing and with an end inspiratory whoop. He was vaccinated according to the National Vaccine Program.

Facing the situation, the child was oriented to our unit in order to exclude tuberculosis. Our health unit is responsible for the diagnosis and management of tuberculosis in our region. On physical examination, the patient had an oral temperature of 37°C and the oxygen saturation was 96% on room air. He was a well-developed, well-nourished young boy with frequent, violent paroxysms of cough. The mucous membranes were moist and the pharynx was slightly injected without exudates. No mass or adenopathy presented on examination of the neck. The lungs presented diffuse crackles and expiratory wheezes. The rest of the physical examination was unremarkable.

**Figure 1:**
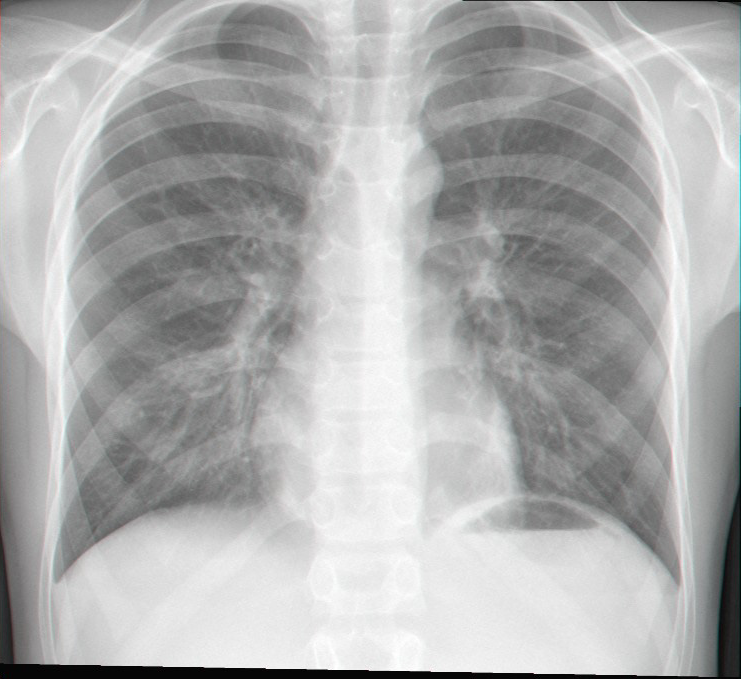
**Chest radiography**.

The blood analysis revealed an increased peripheral white blood cell count with lymphocytosis and a normal biochemistry, including normal C – reactive protein. The chest radiograph showed a reinforcement of the perihilar bonchovascular reticulum and heterogeneous infiltrates on the inferior third of both lung fields (fig. 1).

At this point, the differential diagnosis included viral upper respiratory infection, tuberculosis (as we have an incidence rate of 26.4/100.000 inhabitants) [3] and pertussis. Tuberculin skin test was performed and read 72 hours later and was negative (0 mm). The child was hospitalized, a gastric sample (as he has no sputum) was sent for microbiological study and for detection of *Mycobacterium tuberculosis *and *Bordetella pertussis *deoxyribonucleic acid (DNA) by polymerase chain reaction (PCR) assays.

The PCR analysis of the patient's gastric sample was reported to be positive for *Bordetella pertussis *DNA.

The patient received a prescription of clarithromycin (15 mg/kg/day for 14 days). All those who came in direct contact with the case patient were advised to take a course of clarithromycin as prophylaxis against pertussis infection. The patient's symptoms resolved after two weeks. Two of the patient's family members have developed symptoms of *Bordetella pertussis *infection and were treated after convenient study.

## Discussion

Cough is one of the most common complaints among children and its causes are multiple [4]. This child also presented anorexia and slight weight loss (1, 5 Kg during 4 weeks). Probably it was related to long-lasting clinical symptoms and emetizing cough. In country with tuberculosis incidence of 29/100 000 inhabitants [5], and in the presence of constitutional symptoms, tuberculosis must be excluded. The child soon had weight gain after symptom improvement.

Pertussis, an acute illness of respiratory tract remains endemic in developed nations despite high vaccination coverage [6]. While classical pertussis in the prevacine era was primarily a childhood disease, today with widespread vaccination, there has been a shift in the incidence of disease to adolescents and adults [7]. Indeed, infants are the most vulnerable group with the highest rates of morbidity and mortality, yet adolescents and adults now comprise a significant percentage of cases and a conduit of infection for the infants [7]. PCR, culture and serology are the mainstay of the laboratory diagnosis of pertussis; however in recent years PCR has become an increasingly more popular tool and has significantly contributed to the increasing incidence of pertussis [7]. It is unusual to get positive PCR on sputum specimen 4 weeks after cough start. Actually the sensitivity of PCR decreases with the duration of cough and among previously immunized individuals; nevertheless it is a significantly more robust tool for diagnosis than culture in those in the later stages of disease. A serology test can be a useful tool, particularly among older patients presenting late in the course of their illness when culture and PCR are negative.

In Portugal, pertussis vaccination was introduced by the National Vaccine Program in 1965 and since 1967 the number of reported cases has decreased [8]. In 2004 the number of cases increased, as occurred in other European countries. So, in 2006 the Advisory Committee on Immunization Practices recommended that adolescents between the ages of 11 and 18 should receive another dose of vaccine [9], although in Portugal pertussis vaccination is still provided in five doses of DTaP (2, 4, 6, 18 months and 5–6 years) [7]. Active immunization and early diagnosis are crucial in the management of pertussis.

## Abbreviations

DNA: deoxyribonucleic acid; PCR: polymerase chain reaction

## Competing interests

The authors declare that they have no competing interests.

## Authors' contributions

RD, ACD, LI and NM analyzed and interpreted the patient data. They all gave a major contributor in writing the manuscript. All authors read and approved the final manuscript.

## Consent

Written informed consent was obtained from the patient for publication of this case report and accompanying images. A copy of the written consent is available for review by the Editor-in-Chief of this journal.
